# Potential Associations Between Microbiome and COVID-19

**DOI:** 10.3389/fmed.2021.785496

**Published:** 2021-12-22

**Authors:** Huifen Wang, Haiyu Wang, Ying Sun, Zhigang Ren, Weiwei Zhu, Ang Li, Guangying Cui

**Affiliations:** ^1^Department of Infectious Diseases, The First Affiliated Hospital of Zhengzhou University, Zhengzhou, China; ^2^Gene Hospital of Henan Province, Zhengzhou, China; ^3^Precision Medicine Center, The First Affiliated Hospital of Zhengzhou University, Zhengzhou, China

**Keywords:** COVID-19, microbiota, ACE2, gut-lung axis, probiotics

## Abstract

The coronavirus disease 2019 (COVID-19) pandemic caused by the severe acute respiratory syndrome coronavirus 2 (SARS-CoV-2) has plunged the world into a major crisis. The disease is characterized by strong infectivity, high morbidity, and high mortality. It is still spreading in some countries. Microbiota and their metabolites affect human physiological health and diseases by participating in host digestion and nutrition, promoting metabolic function, and regulating the immune system. Studies have shown that human microecology is associated with many diseases, including COVID-19. In this research, we first reviewed the microbial characteristics of COVID-19 from the aspects of gut microbiome, lung microbime, and oral microbiome. We found that significant changes take place in both the gut microbiome and airway microbiome in patients with COVID-19 and are characterized by an increase in conditional pathogenic bacteria and a decrease in beneficial bacteria. Then, we summarized the possible microecological mechanisms involved in the progression of COVID-19. Intestinal microecological disorders in individuals may be involved in the occurrence and development of COVID-19 in the host through interaction with ACE2, mitochondria, and the lung-gut axis. In addition, fecal bacteria transplantation (FMT), prebiotics, and probiotics may play a positive role in the treatment of COVID-19 and reduce the fatal consequences of the disease.

## Introduction

The novel coronavirus disease 2019 (COVID-19), caused by severe acute respiratory syndrome coronavirus 2 (SARS-CoV-2), is an infectious disease that emerged in the winter of 2019 and typically manifests with systemic symptoms, such as fever, myalgia, and fatigue, respiratory symptoms, including dry cough, dyspnea, and anosmia, and less common symptoms, including aches and pains, nasal congestion, headache, conjunctivitis, sore throat, diarrhea, loss of taste or smell, or a rash on skin or discoloration of fingers or toes (1–3). COVID-19-infected individuals of all ages in nearly every country of the world have a mortality rate of 3.2% (4, 5). As of September 11, 2021, there were more than 155 million confirmed cases of COVID-19 and more than 4.61 million deaths worldwide (Johns Hopkins University Coronavirus Resource Center, 2020, https://coronavirus.jhu.edu/map.html). Currently, there are no specific treatments for COVID-19. In summary, this pandemic has brought great challenges to all humankind. There are still countries that are in extremely brutal situations, such as India (Johns Hopkins University Coronavirus Resource Center, https://coronavirus.jhu.edu/region/india). Thus, a better understanding of COVID-19 is beneficial for the prevention and treatment of the disease.

The human micro-ecosystem is defined as the microbiota living in or on the human body in symbiotic or parasitic relationships, including approximately 1.5–3 kg of microbes. The microbes include bacteria, fungi, viruses, protozoa, and archaeal populations. Referring to the distribution of microbes in humans, they are classified as intestinal micro-ecosystems, oral micro-ecosystems, skin micro-ecosystems, vaginal micro-ecosystems, and so on. Microbiota and their metabolites influence our physiology both in health and disease by participating in host digestion and nutrition, contributing to metabolic functions, modulating the immune system, synthesizing vitamins, and producing a wide variety of biochemically active compounds (6–8). In recent years, the relationship between microbiota and human diseases has been studied extensively. For example, as the largest micro-ecosystem in the human body, the gut micro-ecosystem plays an important role in human health and diseases (9, 10), such as type 2 diabetes (11), obesity (12), autoimmune hepatitis (13), liver cirrhosis (14), early hepatocellular carcinoma (15), and chronic kidney disease (16).

Recently, a large number of microecological studies on COVID-19 have suggested that the micro-ecosystem also plays an important role in COVID-19 and might serve as a novel therapeutic target for improving the prognosis of patients (17, 18). It has been confirmed that there are significant changes in the intestinal and airway microbiomes in patients with COVID-19. The diversity, richness, and uniformity of the airway and intestinal microbiome in patients with SARS-CoV-2 infection were significantly decreased. It is usually characterized by a decrease in beneficial bacteria and an increase in conditional pathogenic bacteria, such as lactic acid bacteria and fecal calcium bacilli, which are negatively correlated with the abundance of the conditional pathogenic bacteria (19–21).

In this study, we summarized the microbial characteristics of COVID-19 and discussed the potential mechanism of microbiota and the therapeutic effects of microbial preparations on the disease. The aim of this study was to reach a better understanding of and improve the prevention and treatment of COVID-19.

## Microbial Characteristics Of Covid-19

### Gut Microbiome

#### Changes in Bacterial Microbiota in COVID-19

After shotgun metagenomic sequencing analyses of fecal samples from patients with COVID-19, Tao z et al. found that the gut microbiome was disturbed, characterized by enrichment of opportunistic pathogens and depletion of beneficial commensals, in patients with COVID-19. The severity of the disease was positively correlated with fecal microbiota alterations, such as the baseline abundance of *Coprobacillus, Clostridium ramosum*, and *Clostridium hathewayi*, but negatively correlated with *Faecalibacterium prausnitzII* (an anti-inflammatory bacterium). *Bacteroides thetaiotaomicron, Bacteroides massiliensis, Bacteroides dorei*, and *Bacteroides ovatus* are inversely correlated with SARS-CoV-2 load in fecal samples from patients, and they downregulate the expression of ACE2 in the murine gut (22). By using 16S ribosomal RNA (rRNA) gene V3-V4 region sequencing to analyze the gut microbiota in patients with COVID-19, patients with H1N1,and healthy controls, it was found that the gut microbiota of COVID-19 patients, while patients with H1N1 changed significantly compared with that of healthy controls (HCs). The bacterial diversity of patients with COVID-19 is usually depressed, accompanied by a lower relative abundance of beneficial symbionts but a higher relative abundance of opportunistic pathogens, such as *Actinomyces, Rothia, Veillonella*, and *Streptococcus*. They identified five bacterial biomarkers (*Intestinibacter, Fusicatenibacter, Actinomyces, Romboutsia*, and *Erysipelatoclostridium*) with high accuracy that could be used to distinguish COVID-19 from HC. The bacterial diversity of patients with H1N1 was lower than that of patients with COVID-19, and the bacterial composition was different. Seven bacterial biomarkers could be used to distinguish the two groups. Changes in the gut microbiome of COVID-19 and H1N1 were correlated with clinical indicators, including WBC, CRP, PCT, D-dimer, IL-2, IL-4, IL-6, and TNF-α (23). In summary, patients with COVID-19 experienced significant changes in their intestinal flora compared to healthy individuals, which is consistent with previous studies on respiratory viral infections (24–27). Older, obese, and comorbid patients are more likely to catch COVID-19 or to develop severe disease after infection (28–31). Patients with diabetes and cardiovascular disease often have a poor prognosis after infection with SARS-CoV-2 (32, 33). There are many different hypotheses regarding the causes of these situations. However, it is undeniable that changes in the gut microbiome are also highly likely to be important influencing factors. The diversity and richness of the gut microbiome decrease with age (34, 35). Obese people and those with other comorbidities also often have changes in their gut microbiome. The reduction of some strains or changes in the structure of the gut microbiome may reduce the body's ability to fight inflammation and regulate the immune system, which may play a negative role in SARS-CoV-2 infection (36–40).

#### Changes in Fungal Microbiomes in COVID-19

A study with 30 patients with COVID-19 studied alterations in the fecal fungal microbiomes (mycobiome) of patients with SARS-CoV-2 infection during hospitalization and upon recovery and found that the fecal mycobiomes of patients with COVID-19 had significant changes compared with the control group. At the time of hospitalization, patients with COVID-19 showed enrichment of *Candida albicans* and a highly heterogeneous mycobiome configuration. In the last test before discharge, the fecal fungal diversity of patients with COVID-19 was 2.5 times higher than that of HCs. Compared with the control group, patients with COVID-19 had an increased proportion of opportunistic fungal pathogens, such as *Candida albicans, Auris candida*, and *Aspergillus flavus*. Even after SARS-CoV-2 was cleared from the nasopharyngeal sample and respiratory symptoms disappeared, *Aspergillus flavus* and *Aspergillus niger* (two respiratory-associated fungal pathogens) were detected in stool samples from a subset of patients with COVID-19. Moreover, up to 12 days after nasopharyngeal clearance of SARS-CoV-2, some patients still had unstable and maladjusted gut mycobiomes (41). The characteristics of the gut microbiome in patients with COVID-19 are shown in Table 1.

**Table 1:** The characteristics of gut microbiome in patients with COVID-19.

	**Sample size**	**Microbial characteristics of COVID-19 patients**	**Corresponding references**
1	Total: 36 (15 COVID-19, 6 Pneumonia controls, 15 Healthy controls)	Enrichment of opportunistic pathogens and depletion of commensals. Baseline fecal abundance of the bacteria *Coprobacillus, Clostridium ramosum, and Clostridium hathewayi* showed significant correlation with COVID-19 severity, whereas an anti-inflammatory bacterium *Faecalibacterium prausnitzii* showed an inverse correlation. Four *Bacteroidetes* members, including *Bacteroides dorei, Bacteroides thetaiotaomicron, Bacteroides massiliensis*, and Bacteroides ovatus, showed significant inverse correlation with fecal SARS-CoV-2 viral load in patients.	(22)
2	Total: 57 hospitalized COVID-19 patients	Significant reduction of probiotic bacteria *Lactobacillus* and *Bifdobacterium*. Significant reduction of anti-inflammatory bacteria (*Butyrate producing bacteria) F. prausnitzii, C. butyricum, C. leptum, E. rectale*.	(26)
3	Total: 15 hospitalized COVID-19 patients	Significant increase of *Collinsella aerofaciens, Collinsella tanakaei, Streptococcus infantis* and *Morganella morganii*. Significant increase of inflammatory and pathogenic bacteria *R. gnavus, Clostridium hathewayi* and *Enterococcus avium* upon clearance of COVID-19 virus from the stool.	(27)
4	Total: 23 6(i-COVID-19); 9 (w-COVID-19), 3 non-COVID-19 hospitalized patients in the ICU, 5 non-COVID-19 patients in general ward	Microbial richness was reduced in i-COVID-19 group compared to the w-COVID-19 group. Signifcant increase of opportunistic pathogens Proteobacteria, *Peptostreptococcaceae, Enterobacteriaceae, Staphylococcaceae, Vibrionaceae, Aerococcaceae, Dermabacteraceae, Actinobacteria* compared to non-COVID-19 patients. Significant decrease of *Spirochaetes and Fusobacteria* compared to non-COVID-19 patients. i-COVID-19 compared to the w-COVID-19, further increase of opportunistic pathogens *Staphylococcaceae, Microbacteriaceae, Micrococcaceae, Pseudonocardiaceae* and *Erysipelotrichales* and other bacteria. Significant decrease of *Carnobacteriaceae, Coriobacteriaceae, Mycoplasmataceae, Pectobacteriaceae, Moritellaceae, Selenomonadaceae*, and *Micromonosporaceae* compared to the w-COVID-19 group.	(20)
5	Total: 69 (30 hospitalized COVID-19 patients, 9 hospitalized CAP patients, 30 healthy individuals)	Significant increase of opportunistic fungal pathogens *C. albicans, Candida auris, Aspergillus favus* and *Aspergillus niger* in COVID-19 group Similar heterogeneous mycobiome composition found in the community-acquired pneumonia group.	(41)
6	Total: 84 (30 hospitalized COVID-19 patients, 24 hospitalized H1N1 patients, 30 healthy individuals)	Microbial profles among COVID-19 and H1N1 patients were significantly less diversified than the control group H1N1 group Butyrate-producing bacteria *Lachnospiraceae, Ruminococcaceae, Blautia, Agathobacter, Anaerostipes, Fusicatenibacter, Eubacterium halii, Dorea, Faecalibacterium s*ignifcantly decreased compared to the healthy control group. COVID-19 group Depletion of *Fusicatenibacter, Romboutsia, Anaerostipes, Eubacterium hallii, Ruminococcus torques* and *Blautia* in COVID-19 patients compared to the healthy control group. Higher abundance of *Streptococcus, Fusicatenibacter, Collinsella, Dorea, Agathobacter, Eubacterium hallii, Ruminococcus torques* in COVID-19 subjects compared to H1N1 group.	(23)

*(i-COVID19), COVID-19 patients in the ICU; (w-COVID-19), COVID-19 patients in the infectious disease wards*.

### Lung Microbiome

A study found that the lung microbiota in patients with COVID-19 and patients with community-acquired pneumonia (CAP) was significantly different from that in healthy people by analyzing bronchoalveolar lavage fluid (BALF). The microbial composition of patients infected with SARS-CoV-2 was similar to that of patients with CAP. However, this study enrolled only eight patients with COVID-19. The small sample size limits these research results (42). As nasopharyngeal swabs can be a reasonable proxy for lung samples, some studies focus on nasopharyngeal bacterial communities. According to the bacterial communities of the nasopharynx in mild COVID-19, no statistically significant differences were found in either bacterial richness and diversity or composition, indicating that nasopharyngeal microbiota might be resilient to COVID-19 at least in the early stages (43). It can be inferred that the lung bacterial community, like nasopharynx microbiota, can also protect against SARS-CoV-2 growth or attachment in the host (44). Rueca M et al. found that the complete disappearance of Bifidobacterium and Clostridium is characteristic of the nasal/oropharyngeal microbiological spectrum in patients with COVID-19 treated in the ICU, while Salmonella, Scardovia, Serratia, and fruit bacilli are only detected in ICU SARS-CoV-2 patients (45). This finding may be beneficial to the stratification of patients with SARS-CoV-2.

### Oral Microbiome

The oral microbiome is composed of more than 600 endemic groups and can be divided into subgroups (46, 47). There is an oral microbial imbalance in patients with COVID-19. One study explored oral microbiome changes in patients with COVID-19 and recovered patients. The study included 392 tongue coating samples from central and eastern China. The results showed that oral microbial diversity was significantly lower in the CPS (confirmed patients) group than in the healthy control (HC). Compared with HC, there were fewer butyric acid-producing bacteria and more lipopolysaccharide-producing bacteria in the CPS group. The classifiers based on eight optimal oral microbial markers (7 fecal microbial markers) had good diagnostic efficiency in different chemotypes. Importantly, the diagnostic response in the cross-regional cohort was 87.24% (19).

In addition, *Fusobacterium periodonticum* may increase susceptibility to COVID-19 infection. Compared to the control, *Firmicutes, Bacteroidetes, and Actinobacteria* increased in the COVID-19 group, and *Proteobacteria, Fusobacteria, Leptotrichia*, and *Haemophilus* decreased. Moreover, Fusobacterium decreased further in patients with COVID-19 with severe illness. Similarly, other studies have found changes (48) in the composition of oral microflora in patients (48–50). The characteristics of the airway microbiome in patients with COVID-19 are shown in Table 2.

**Table 2:** The characteristics of airway microbiome in patients with COVID-19.

	**Sample size**	**Microbial characteristics of COVID-19 patients**	**Corresponding references**
1	Total: 957 (496 tongue-coating samples, 226 fecal samples and 235 serum samples)	Oral microbial diversity was significantly decreased in CPs vs. healthy controls (HCs). Compared with HCs, butyric acid-producing bacteria were decreased and lipopolysaccharide producing bacteria were increased in CPs in oral cavity.	(19)
2	Total: 74 (19 mild COVID-19 18 severe COVID-19, 19 critical COVID-19 patents, 18 COVID-19 negative individuals)	Signifcant increase of *Firmicutes, Bacteroidota, Proteobacteria, Actinobacteria* in covid-19 patients compared to non-COVID-19 patients. Signifcant increase of opportunistic pathogens *Streptococcus, Prevotella, Veillonella, Haemophilus, Moraxella and Leptotrichia* in covid-19 patients compared to non-COVID-19 patients. Higher abundance of *Prevotella* was found in more severe COVID-19 patients compared to less severe COVID-19 patients.	(50)
3	Total: 39 (10 COVID-19 ICU patients, 11 mild to moderate COVID-19 patients, 8 other coronaviruses patients, 10 healthy individuals)	The nasal/oropharyngeal microbiota profiles of SARS-CoV-2 patients admitted to ICU are characterized by a complete depletion of *Bifidobacterium and Clostridium*, while *Salmonella, Scardovia, Serratia* and *Pectobacteriaceae* were exclusively detected in ICU SARS-CoV-2 patients.	(45)
4	Total: 187 (62 COVID-19 patients, 125 non-COVID pneumonia patients)	Airway microbiome in COVID-19 samples were less diversifed. Certain microbiota were associated with CRP concentration 47.4% of COVID-19 samples revealed an increase of presence in opportunistic pathogens compared to 52% of non-COVID-19 samples. Increased abundance of Human infuenza virus, Respiratory syncytial viruses, Human alphaherpesvirus 1 and *Candida albicans* in COVID-19 patients compared to non-COVID-19 patients.	(48)
5	Total: 38 (18 COVID-19 patients, 8 recovered COVID-19 patients, 12 healthy individuals)	*Fusobacterium periodonticum may increase the susceptibility to COVID-19 infection*. Significant increase of *Firmicutes, Bacteroidetes, Actinobacteria* in COVID-19 group compared to control group. Significant decrease of *Proteobacteria, Fusobacteria, Leptotrichia and Haemophilus* compared to A further reduction of *Fusobacterium* was reported in more severe patients compared to less severe COVID-19 patients.	(49)
6	Total: 64 (35 COVID-19 patients, 10 non-COVID-19 patients with other diseases, 19 healthy subjects)	The diversity, richness, and evenness of airway microbiome was significantly lower in COVID-19 patients compared to healthy control group. *Faecalibacterium* were negatively correlated with the abundance of opportunistic pathogen. Significant increase of opportunistic pathogens *Rothia, Porphyromonas, Fusobacterium, Neisseria*, and *Saccharibacteria incertae sedis i*n the airway of COVID-19 patients compared to healthy control.	(21)

## Microbiota Mechanism Of Action On Covid-19

### The Intestinal Microecological Disturbance in Patients With COVID-19 May Be Closely Related to ACE2

RNA shotgun metagenomics sequencing was used on serial fecal viral extractions from 15 hospitalized patients with COVID-19; some of these patients infected with SARS-CoV-2 had an active gut viral infection even though they did not exhibit gastrointestinal (GI) symptoms (46.7%). The virus still exhibited high transcriptional activity and replication in the gut after SARS-CoV-2 was cleared from the respiratory system. In patients with COVID-19 with a high level of SARS-CoV-2 infectivity, opportunistic pathogens had a higher abundance in the gut, such as *Streptococcus infantis, Morganella morganii, Collinsella aerofaciens*, and *Collinsella tanakaei*. These bacteria usually have high functions of nucleotide ab initio synthesis, amino acid biosynthesis, and glycolysis. In contrast, low- to no SARS-CoV-2 infectivity patients are enriched with more short-chain fatty acid-producing bacteria, *Lachnospiraceae bacterium 1_1_57FAA, Alistipes onderdonkii, Parabacteroides merdae*, and *Bacteroides stercoris* (27). A study found that the infectious SARS-CoV-2 virus was isolated from the stool sample of a patient with COVID-19 (51). These findings provide evidence for active or quiescent gastrointestinal infections associated with SARS-CoV-2.

Angiotensin-converting enzyme 2 (ACE2) is widely expressed in the gut, kidneys, lungs, cardiovascular system, central nervous system, and adipose tissue. ACE2 is known as the negative regulator of the renin-angiotensin system and the promoter of amino acid transport. Recently, it has also been found to be the receptor of severe acute respiratory syndrome coronavirus (SARS-CoV) and SARS-CoV-2 (52–54). Although respiratory symptoms and lung injury are the main manifestations of SARS-CoV-2 infection, the myocardium, kidney, intestine, and liver are also damaged in patients with COVID-19 (55, 56). Therefore, patients with COVID-19 have multiple organ dysfunction, in addition to respiratory system involvement (57–59).

Viral S protein has been identified as an important determinant of host tropism. SARS-CoV-2 can use a variety of host proteases to modify the s protein and ACE2, promoting the binding of the S protein and promoting receptors (60, 61). The binding affinity between SARS-CoV-2 and ACE2 seems to be stronger than that of SARS-CoV (62, 63). ACE2 is widely expressed in intestinal epithelial cells, so it may be the binding site of intestinal SARS-CoV-2 infection. Previous experiments have also confirmed that gastrointestinal leakage can be improved and worsened with an increase or decrease in ACE2. Gut microecological disorders in patients with COVID-19 may be closely related to the loss of ACE2 and the overactivation of the ACE/AngII/AT1R (angiotensin II receptor type 1) axis (64).

A previous study found that germ-free (GF) mice deteriorate colitis after accepting Ace2^−/−^ gut microbiota. Reshaping the gut microbiological composition of Ace2^−/−^ mice can increase susceptibility to intestinal inflammation. The results confirmed that ACE2 plays an important role in regulating amino acid homeostasis, innate immunity, intestinal microbial ecology, and susceptibility to colitis (65). A study on rats found that compared with GF (germ-free) rats, the expression of ACE2 in conventionalized GF (GFC) rats was significantly decreased, and the intensity of systemic inflammation was increased, which was characterized by a significant increase in the level of lipoprotein troponin 2 (Lcn2). The levels of tryptophan metabolites, canine uric acid, and hydroxycanine in GFC rats were higher. These results suggest that the intestinal microflora is a key factor in regulating the expression of ACE2 in the colon. These factors may lead to intestinal-lung pathological changes during the period of COVID-19 (66). After SARS-CoV-2 infection, the protective function of ACE2 is lost, leading to impaired RAS signaling, which exacerbates inflammatory phenotypes, aggravates the systemic “cytokine storm” and damages tissue (67–69). The change in intestinal microbiota, altered intestinal barrier permeability, and subsequent inefficient initiation of local and systemic immunity may be amplified by loss of the ACE2 protective function. A study by Zang et al. confirmed this hypothesis, clearly demonstrating that SARS-CoV-2 is capable of infecting ACE2^+^ mature intestinal cells in the human small intestine mediated by TMPRSS2 and TMPRSS4 proteases (70). SARS-CoV-2 infection may significantly reduce intestinal ACE2 expression, lead to leakage of the intestinal barrier, alter microbiota composition and metabolome, and aggravate endotoxemia/inflammation. In support of these possibilities, recent data suggest that some patients with COVID-19 have intestinal microbiome disorders (71).

### The Relationship Between Mitochondria, Intestinal Microorganisms, and COVID-19

Mitochondria are the energy-producing structures or powerhouses in cells and the main location of aerobic respiration. They have a variety of interrelated functions. They not only produce ATP and participate in biosynthesis but also promote cell stress responses, such as autophagy and apoptosis. Mitochondria are intimately connected to other cellular compartments, and they form a dynamic, complex, and interconnected network (72, 73).

It has been found that the interaction between microflora and host cells affects human health *via* the mitochondria. There is a correlation between the quality and diversity of microflora and mitochondrial function. Reactive oxygen species (ROS) produced by mitochondria participate in the innate immune response and inflammatory process as targets of pathogens. The data show that the excessive production of mitochondrial ROS may affect the ROS signal transduction induced by the microflora, thus regulating the intestinal epithelial barrier. At the same time, the metabolites released by the microflora can directly interfere with the production of the mitochondrial respiratory chain and ATP. These results indicate that the microbiota targets mitochondria to influence the host and maintain balance. Loss of this balance can lead to illness, such as colitis (74).

It has been reported that SARS-CoV-2 can colonize the gastrointestinal tract and thus interfere with the intestinal microbiome (75). Gut microbiota composition may be a susceptibility factor for COVID-19. Gut microbial features have a high correlation with blood proteomic biomarkers of severe COVID-19. Changes in gut microbiome features may lead to abnormal inflammatory status and further reduce resistance to SARS-CoV-2 infection. There are individual differences in the composition of people's microbiota. Therefore, in the global onset of COVID-19, different populations had different susceptibilities to the disease. Mitochondria can exacerbate the body's inflammatory response by regulating immune responses when the body is stimulated by a virus (76). Mitochondria can also cause changes in gut microbiome characteristics. The microflora can act on the host with mitochondria as the target. There is an interaction between the microbiome and mitochondria, and this interaction appears to be linked primarily through endocrine, immune, and humoral pathways (74, 77–79).

In addition, SCFAs such as n-butyric acid of intestinal symbiotic flora can reduce oxidative stress and ROS production. This may further affect the regulation of the intestinal barrier. Interestingly, mitochondrial ROS may also be involved in the regulation of the intestinal epithelial barrier. Mitochondria release proteins or nucleotides that activate formyl peptide receptors (FPRs). Mitochondrial dysfunction induced by the use of dinitrophenol affects epithelial barrier dysfunction (80). SARS-CoV-2 has been reported to affect the gastrointestinal system and the intestinal barrier, and subsequent disruption of barrier function or intestinal microbial disturbance may affect the progression and severity of the virus (75). Mitochondria are also one of the targets of iron oxidation stress in the body, and the absorption of iron, that is, the storage of ferritin in online granules, contributes to the normal function of mitochondria. On the contrary, the imbalance of iron metabolism affects the normal function of mitochondria (81). However, studies have confirmed that systemic methemoglobinemia in patients with COVID-19 is associated with the severity of the disease and poor prognosis. Methemoglobinemia can also lead to increased oxidative stress and mitochondrial dysfunction. The aerobic respiration of mitochondria is blocked, and a large number of Inflammatory cytokines and ROS is produced, which further aggravates the inflammation of the body (82–84). Through this analysis, we can conclude that there is also a strong correlation between gut microbiota and mitochondrial function in COVID-19 disease. Mitochondria may participate in the pathogenesis of COVID-19 by mediating the production of ROS. The complex interactions between mitochondrial dysfunction, oxidative stress, and gut microbiome changes and the roles they play in the pathogenesis of COVID-19 require further investigation.

### The Gut-Lung Axis Is Associated With COVID-19 Severity, and the Microbial Imbalance

**Figure 1:**
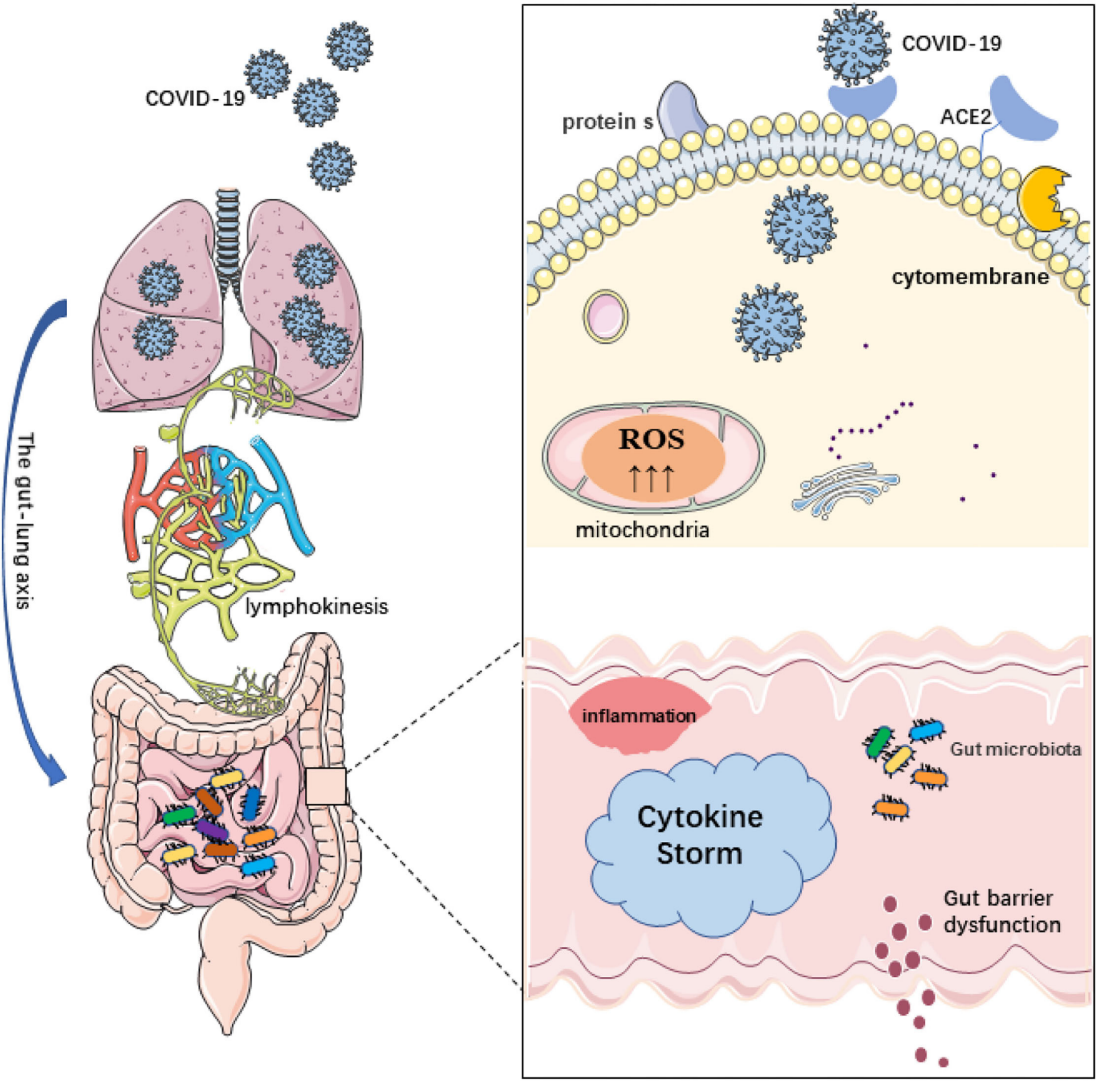
Schematic diagram of coronavirus disease 2019 (COVID-19) infection and its relationship to the gut-lung axis and microbiome disorders. 1. The destruction of the intestinal barrier integrity by microbial imbalance may lead to the migration of severe acute respiratory syndrome coronavirus 2 (SARS-CoV-2) from the lungs through the circulatory and lymphatic systems to the intestinal cavity. Conversely, bacterial disorders can lead to the rupture of the intestinal barrier and the migration of SARS-CoV-2 viruses from the intestines to the lungs. 2. ACE2 is widely expressed in intestinal epithelial cells. SARS-CoV-2 can use a variety of host proteases to modify the s protein and ACE2 to promote the binding of SARS-CoV-2 to the receptor. After COVID-19 infection, the protective function of ACE2 is lost, which leads to damage of the RAS signal, aggravation of the inflammatory phenotype, and further aggravation of the systemic “cytokine storm” and tissue damage. The permeability of the intestinal barrier changes and intestinal leakage even occurs. 3. In addition, reactive oxygen species (ROS) produced by mitochondria are involved in the innate immune response and inflammation as targets of pathogens. The excessive production of mitochondrial ROS may affect the ROS signal transduction induced by the flora and regulate intestinal barrier function.

Gut-lung axis also plays a crucial role in COVID-19. The gut-lung axis is associated with the severity of the disease and with microbial imbalance. Damage to the integrity of the intestinal barrier due to microbial imbalance may cause SARS-CoV-2 to migrate from the lungs to the intestinal lumen through the circulatory and lymphatic systems. This may explain the gastrointestinal symptoms in patients with COVID-19. In addition, a decrease in the diversity of intestinal microflora with age may account for the higher risk of severe COVID-19 in older persons. Given the studies to date on coronavirus and the relationship between the intestinal microflora and the immune system, further study of the entero-lung axis and its relationship with the severity of COVID-19 is needed. Dysbacteriosis can cause intestinal barriers to break down and SARS-CoV-2 viruses to migrate from the intestines to the lungs (85). It can be said that the gut-lung axis is involved in the immune response to SARS-CoV-2. Intestinal malnutrition is associated with increased mortality from other respiratory infections due to increased inflammation in the lungs and intestines and reduced regulatory or anti-inflammatory mechanisms (86). This is actually a phenomenon of organ crosstalk, because organs in different parts may develop from the same germ layer during early development. In addition, from an immune point of view, it has been confirmed that there is a cross effect between the intestinal mucosa and the mucosa of the respiratory system. The gastrointestinal symptoms of patients with COVID-19 are the key that cannot be ignored. The intestinal damage caused by this respiratory disease may not be caused by intestinal infection but may be caused by a pulmonary infection which is used in the intestinal tract through the action of the gut-lung axis. And the lungs have also been confirmed to have the existence of bacteria and participate in the immune response of the body (87, 88). Some of the patients with COVID-19 died of acute respiratory disease syndrome (ARDS). In these patients, the imbalance of intestinal flora is also more serious. It is suggested that the interaction between intestines and lungs may regulate the inflammatory response of patients with COVID-19 through the gut-lung axis (89, 90). Although the conclusions of these studies are mostly descriptive and the exact pathogenesis of COVID-19 is not completely clear, these conclusions provide important evidence and direction for future research and lay a foundation for potential treatment strategies. COVID-19 infection and its relationship to the gut-lung axis and microbiome disorders are shown in Figure 1.

## Expectation

### The Microbiome Is Involved in the Diagnosis and Treatment of COVID-19 Disease

As mentioned earlier, the evidence shows that the diversity and richness of the COVID-19 patient's microflora change, and this change is related to some clinical indicators (23). Individuals have different susceptibilities to the SARS-CoV-2 virus. Therefore, we can speculate that detecting the microflora of patients is a way to help diagnose viral infections and predict susceptible populations (24, 25).

Dickson et al. found gut-related Bacteroides in the BALF of many patients with acute respiratory distress syndrome (ARDS) by high-throughput sequencing, which is rare in healthy controls, and the enrichment of these pulmonary bacteria is related to the increase in inflammatory markers in plasma (91). Another study found that the main characteristics of the lung microbiome (bacterial load and enrichment of intestinal-associated bacteria) predicted the prognosis of critically ill patients. Through the detection of 91 critically ill patients, it was found that the patients with increased bacterial burden in the lungs had fewer days off ventilators, and the composition of the bacterial community in the lungs predicted the days without ventilators, which was driven by the existence of intestinal-related bacteria (such as *Lachnospiraceae* and *Enterobacteriaceae*). The increase in intestinal-associated bacteria is a strong predictor of decreased survival in patients with ARDS (92). ARDS is a common and serious complication of COVID-19, and it is an important factor leading to the death of critically ill patients. There is evidence that the composition of pulmonary flora in patients with ARDS changes. Therefore, changes in the composition of microorganisms in the lungs of patients with COVID-19 may predict the occurrence of ARDS (92–96). In addition, improving the microflora of patients and promoting the balance of gut microecology will be beneficial in the treatment of patients with COVID-19; that is, gut microflora can be used as a therapeutic target of pneumonia.

### The Hypothesis of FMT for COVID-19 Therapy

Microbiome-based therapeutics, as a new way to treat diseases, have promising prospects in clinical application (97). Research has shown that fecal microbiome transplantation or fecal microbiota transplantation (FMT) has therapeutic effects in many diseases (98, 99). J. K et al. found that fecal microbiota transplantation can restore mucosal integrity and bacterial levels in a murine model of burn injury (100). It has been reported that after FMT, multiple organ dysfunction syndrome (MODS) patients' symptoms and diarrhea were significantly relieved. Defecation and body temperature returned to normal (101). A case report described the procedure using FMT to treat a patient with sepsis and severe diarrhea after vagotomy (102). As mentioned earlier, the gut-lung axis can maintain host homeostasis and disease development with the association of the immune system (88, 103). The gut-lung axis may influence the patient's systemic inflammation. Modulating the gut microbiota composition may play a role in inflammation control in COVID-19 (18). A prospective, interventional search has studied the role of FMT in patients with COVID-19. A total of 11 inpatients with COVID-19 were recruited for this study. All subjects took FMT capsules orally for 4 days, 10 tablets a day. After FMT, gastrointestinal symptoms were improved in five of 11 patients. Peripheral blood lymphocyte subsets changed, and microbial community richness increased. At the gate level, FMT can partially restore intestinal ecological disorders by increasing the relative abundance of Actinobacteria (15%) and reducing the relative abundance of Proteobacteria (2.8%). At the genus level, Bifidobacterium and Faecalibacterium increased significantly. Therefore, FMT may become an intervention for COVID-19.

### Probiotics and Prebiotics Are Expected to Be Important Drugs for the Treatment of COVID-19 by Regulating Micro Ecological Balance

Probiotics are active microorganisms that are beneficial to the host by colonizing the human body and changing the flora composition of a certain part of the host. Strains belonging to *Bifidobacterium* and *Lactobacillus* are the predominant and subdominant groups of the gastrointestinal microbiota, respectively (104). Currently, the role of probiotics in a variety of diseases has been reported. Probiotics have shown some therapeutic effects in respiratory diseases. A randomized controlled trial found that fermented dairy products containing the probiotic *Lactobacillus casei* DN-114001 can reduce the duration of respiratory tract infection in middle-aged and elderly people (105). At least 3 months after healthy people consumed *Lactobacillus gasseri* PA 16/8, *Bifidobacterium longum* SP 07/3, *B. bifidum* MF 20/5 (5 × 10^7^ cfu/tablet), the incidence of colds and the duration of cold symptoms, such as fever, decreased. Some probiotics, such as *bifidobacteria* or *lactobacilli*, may have an effect on the clearance of influenza viruses from the respiratory tract. This has been demonstrated in humans and mice (88, 106–108). Probiotics can affect the lung immune coagulation induced by TLR3 activation. This process occurs by regulating the production of inflammatory cytokines and the expression of tissue factors and thrombomodulin in the lungs. This study also proved that the prophylactic treatment of probiotics can effectively regulate the balance between respiratory viruses and the control of immune coagulation reaction so that the normal function can resist the viral attack (109).

Some patients with COVID-19 suddenly worsen and develop ARDS. Once ARDS occurs, it easily causes shock and tissue perfusion disorder, and patients often die of multiple organ failure, mainly caused by an immunologic response that is provoked by the infection (known as the cytokine storm) (110). Therefore, if probiotics can be used to assist in the treatment of COVID-19, it is likely to reduce the inflammatory state caused by the immune response and reduce the occurrence of complications.

In terms of the gut-lung axis, the role of probiotics is also worth studying. It has been reported that suckling rats can be protected from rotavirus gastroenteritis by giving them *Bifidobacterium* breve M-16V (PRO) (111). Probiotics may repair intestinal barriers. *Escherichia coli Nissle* 1,917 participates in ZO-2 and PKC zeta redistribution, increasing tight junctions and epithelial barrier repair (112, 113). As mentioned above, COVID-19 is associated with an imbalance of gut microbiota (17). Some probiotics can restore the intestinal micro ecological balance. A study reported that *L. fermentum* CECT5716 or *L. salivarius* CECT5713 can be used to treat infectious mastitis during lactation (114). We see that oral probiotics can play a role by migrating from one part of the body to another. In this way, the use of certain probiotics can improve the microecological balance through the gut-lung axis and thus combat COVID-19. Of course, more research and clinical trials are needed to explore this problem and obtain the most reliable conclusions.

Prebiotics refer to some organic substances that are not digested and absorbed by the host but can selectively promote the metabolism and proliferation of beneficial bacteria in the body to improve the health of the host. When prebiotics passes through the upper digestive tract, most of them are not digested and can be fermented by intestinal flora. Prebiotics only stimulate the growth of beneficial flora, not harmful bacteria with potentially pathogenic or decaying activity (115, 116). After a review of 12 studies investigating the effects of prebiotics and probiotic supplementation on influenza infection, it was concluded that probiotic and prebiotic supplementation could increase the titer of hemagglutination inhibition antibodies after influenza vaccination. In addition, prebiotics may have a direct effect on GI symptoms caused by COVID-19 by blocking ACE enzymes, and ACE2 is thought to be a gateway for SARS-CoV-2 to bind and attack the body. Therefore, by improving the growth and survival ability of probiotics, prebiotics may also have excellent potential against COVID-19 (117, 118). The use of probiotics in clinical treatment for COVID-19 may improve patients' lung and intestinal microbiota and enhance immunity. That may be an appropriate strategy. The treatment plan of the latest edition (eighth edition) in China mentions the use of intestinal microecological regulators to maintain intestinal micro ecological balance and prevent secondary bacterial infection. The balance of gut microflora is an important point in the treatment of “four-resistance and two-balance” in the treatment of critically ill patients, as advocated by academician Li Lanjuan. Although these findings emphasize the adjustment of microecological balance to reduce secondary infection caused by bacterial translocation, the role of gut microflora balance should not be underestimated because of the correlation between intestinal microflora composition and COVID-19.

## Conclusion

In this review, we expounded on the relationship between the microbiota and COVID-19 from the aspects of characteristics, pathogenesis, and treatment. Thus far, we can come to several conclusions. First, the composition of the microbiome of patients with COVID-19 changes significantly compared to that of normal people, particularly gut and lung microbiota. This suggests that the microbiota can be used as a marker to predict the prognosis of ARDS and COVID-19. Then, the gut microbiota can regulate the expression of ACE2 in the intestinal tract, which is the receptor of SARS-CoV-2. The gut-lung axis also plays a very important role in COVID-19. Regardless of FMT or oral probiotics, regulation of gut microbiota dysregulation may reduce inflammation or complications associated with COVID-19, leading to new breakthroughs in the prevention and treatment of the disease.

To date, the COVID-19 pandemic continues to affect people's health and lives. Therefore, studies on the pathogenesis of COVID-19, the search for new treatment methods, the discovery of specific therapeutic drugs, and the development of vaccines are still crucial to prevent and treat the disease. Although the relationship between microbiota and COVID-19 diseases has been extensively studied, the conclusions in terms of pathogenesis and prevention and treatment are mostly descriptive, which is insufficient. These conclusions undoubtedly open up new directions for the prevention and treatment of COVID-19. This promising direction requires further study to reach a more definitive conclusion. In addition, most of the current studies on COVID-19 and microbiota focus on the relationship between gut microbiota and COVID-19, while oral microbiota, as another major direction in the field of microecology, has rarely been reported. On the one hand, the intestinal microbiome is relatively stable, and the relationship between the intestinal microbiome and disease has been studied by many scholars; on the other hand, the relationship between COVID-19 and the digestive tract is very close. Many patients have gastrointestinal symptoms, and given the existence of the lung-gut axis, the need to explore the relationship between COVID-19 and the intestinal microbiome is obvious. Likewise, the COVID-19-induced changes in respiratory tract microorganisms are an obvious subject for further research.

Therefore, for COVID-19, an infectious disease transmitted by the respiratory tract, the relationship between the oral microbiota and COVID-19 is worth studying.

## Author Contributions

GC and AL designed the study. HuW, HaW, YS, and WZ collected the data. HuW and AL analyzed the data. HuW, ZR, and GC wrote the manuscript. All authors reviewed and approved the manuscript.

## Funding

This study was sponsored by grants from National Key Research and Development Program of China (2018YFC2000501) and National Natural Science Foundation of China (U2004121, 82070643, and U1904164).

## Conflict of Interest

The authors declare that the research was conducted in the absence of any commercial or financial relationships that could be construed as a potential conflict of interest.

## Publisher's Note

All claims expressed in this article are solely those of the authors and do not necessarily represent those of their affiliated organizations, or those of the publisher, the editors and the reviewers. Any product that may be evaluated in this article, or claim that may be made by its manufacturer, is not guaranteed or endorsed by the publisher.
